# Comparative proteomics analysis of Neisseria gonorrhoeae strains in response to extended-spectrum cephalosporins

**DOI:** 10.17179/excli2017-832

**Published:** 2017-11-08

**Authors:** Sunanta Nabu, Ratana Lawung, Patcharee Isarankura-Na-Ayudhya, Sittiruk Roytrakul, Supamas Dolprasit, Sineenart Sengyee, Chartchalerm Isarankura-Na-Ayudhya, Virapong Prachayasittikul

**Affiliations:** 1 Department of Clinical Microbiology and Applied Technology, Faculty of Medical Technology, Mahidol University, Bangkok 10700, Thailand; 2 Center of Medical Laboratory Services, Faculty of Medical Technology, Mahidol University, Bangkok 10700, Thailand; 3 Department of Medical Technology, Faculty of Allied Health Science, Thammasat University, Pathumthani 12120, Thailand; 4 Genome Institute, National Center for Genetic Engineering and Biotechnology, National Science and Technology Development Agency, Pathumthani 12120, Thailand

**Keywords:** Neisseria gonorrhoeae, extended-spectrum cephalosporins, antimicrobial resistance, proteomics

## Abstract

*Neisseria gonorrhoeae *strains displaying reduced susceptibility and resistance to extended-spectrum cephalosporins (ESCs) are major public health concerns. Although resistance mechanisms of ESCs have extensively been studied, the proteome-wide investigation on the biological response to the antibiotic stress is still limited. Herein, a proteomics approach based on two-dimensional gel electrophoresis and MALDI-TOF/TOF-MS analysis was applied to investigate the global protein expression under ESC stresses of ESC-susceptible and ESC-reduced susceptible *N. gonorrhoeae* strains. Upon exposure to ceftriaxone, 14 and 21 proteins of ESC-susceptible and ESC-reduced susceptible strains, respectively, were shown to be differentially expressed. In the meanwhile, differential expressions of 13 and 17 proteins were detected under cefixime stress for ESC-susceptible and ESC-reduced susceptible strains, respectively. ESC antibiotics have been proven to trigger the expression of several proteins implicated in a variety of biological functions including transport system, energy metabolism, stress response and pathogenic virulence factors. Interestingly, macrophage infectivity potentiators (Ng-MIP) showed increased expression for ESC-reduced susceptible strain under ESC stress. The altered expression of Ng-MIP was found to be a unique response to ESC stresses. Our finding proposes a broad view on proteomic changes in *N. gonorrhoeae *in response to ESC antibiotics that provides further insights into the gonococcal antimicrobial resistance and physiological adaptation mechanism.

## Introduction

*Neisseria gonorrhoeae*, a causative agent of gonorrhoea, is the second most common curable sexually transmitted infections (STIs) (WHO, 2012[65]; Unemo et al., 2017[56]). *N. gonorrhoeae *infection becomes a global public health problem owing to high prevalence rates as well as the rapid and progressive development of antimicrobial resistance (AMR) to all previously and currently recommended antibiotics available for treatment (WHO, 2016[66]; Alirol et al., 2017[2]). Importantly, extended-spectrum cephalosporins (ESCs) (e.g. oral cefixime and injectable ceftriaxone) are recommended as the first-line antimicrobial monotherapy for gonorrhoea in most settings and areas (Center for Disease Control and Prevention, 2010[8], 2012[9]; Bignell and Unemo, 2013[6]; WHO, 2012[65], 2016[66]; Tapsall, 2009[51]). Unfortunately, treatment failures of ESCs together with the emergence of *N. gonorrhoeae *displaying decreased susceptibility or resistance to ESCs are increasingly being reported worldwide (Ameyama et al., 2002[3]; Ison et al., 2011[25]; WHO, 2014[64]; Unemo et al., 2010[59], 2011[57][58]). The widespread of *N. gonorrhoeae* with decreased susceptibility/resistance to ESCs together with the absence of new effective treatment options for gonorrhoea forewarns a future era of untreatable gonorrhoea. Thus, it is essential to elucidate resistance mechanisms, search for novel target proteins and develop novel antimicrobial agents for effective treatment of gonorrhoea.

Mechanisms of decreased susceptibility/resistance to ESCs in *N. gonorrhoeae* have previously been investigated. The main mechanism involves the alteration of *penA* gene, which encodes the penicillin binding protein 2 (PBP2) that is considered to be a lethal target for β-lactams. Mosaic PBP2 and non-mosaic PBP2 are associated with increased minimum inhibitory concentrations (MICs) of ESCs (Abrams et al., 2017[1]; Peng et al., 2017[41]; Zhao et al., 2017[70]). In addition, the overexpression of the MtrC-MtrD-MtrE efflux pump (i.e. arising from the mutation in *mtrR*) in combination with the alteration of porin PorB1b (i.e. involving *penB*) and an unknown resistance determinant, termed “Factor X”, have been described for increasing the MIC level of ESCs (Lee et al., 2010[31]; Liao et al., 2011[33]; Lindberg et al., 2007[36]; Tanaka et al., 2006[50]; Zhao et al., 2017[70]; Zhao et al., 2009[71]).

Although resistance mechanisms of ESCs have been intensely studied, a comprehensive understanding on the resistance mechanism, the physiological adaptation and compensation mechanisms to antibiotic stress are quite limited. Recent studies have demonstrated that antibiotics affect several biological processes, which is in addition to its target protein, along with species-specific and antibiotic-specific response (Fajardo and Martínez, 2008[18]; Kohanski et al., 2010[28]; Linares et al., 2006[35]). Such knowledge leads to the discovery of novel targets and, in turn, the development of novel antibiotics. In achieving a broad understanding on many facets of antibiotic research, global approaches (e.g. whole genome sequencing, DNA microarray, transcriptomics and proteomics) have become instrumental for studying the mode of action of antibiotics and for the discovery of both primary and secondary resistance mechanisms as well as the physiological adaptation and the maintenance of resistant bacteria (Cao et al., 2015[7]; Eyre et al., 2017[17]; Harrison et al., 2017[20]; Lima et al., 2013[34]).

Therefore, cellular responses of ESC-susceptible and ESC-reduced susceptible *N. gonorrhoeae* against sub-minimum inhibitory concentrations (sub-MICs) of cefixime and ceftriaxone have been elucidated using two-dimensional gel electrophoresis (2-DE) and MALDI-TOF/TOF-MS analysis. The global protein expression of *N. gonorrhoeae* involved in bacterial survival as well as cellular adaptation against ECS stresses are explored herein.

## Materials and Methods

### N. gonorrhoeae strains and culture condition

*N. gonorrhoeae* strain ATCC 49226 and clinical isolates were kindly provided by the National Center of Sexually Transmitted Diseases, Bangrak Hospital, Bangkok, Thailand. *N. gonorrhoeae* isolates from frozen stock were grown on chocolate agar and incubated at 37 °C in 5 % CO_2 _overnight. Next, bacteria were sub-cultivated on chocolate agar at 37 °C in 5 % CO_2_ for 18 h. Colonies of *N. gonorrhoeae* were re-suspended in 100 ml of pre-warmed GC broth (GCB) (1.5 % Proteose Peptone no. 3 [Difco], 0.4 % K_2_HPO_4_, 0.1 % KH_2_PO_4_, 0.1 % NaCl, 0.042 % NaHCO_3_ and 1 % define growth supplement) to an initial optical density at 600 nm of approximately 0.08 for experimental cultures containing 0.25x, 0.5x and 1x MICs of cefixime or ceftriaxone while control cultures were grown without antibiotics. Cultures were continuously incubated on a shaking incubator at 180 rpm at 37 °C for approximately 8 h. For growth rate measurement, aliquots of culture were subjected to measurement of the turbidity at OD_600_ for every 1 h.

### Antimicrobial susceptibility testing

The MIC was determined by an agar dilution method according to guidelines of the Clinical and Laboratory Standards Institute (2014[12]). Antimicrobial agents used in this study include penicillin G (Bio Basic Inc.), cefixime (Fluka), ceftriaxone (Sigma), ciprofloxacin (Fluka), tetracycline (Bio Basic Inc.), azithromycin (Fluka) and spectinomycin (Bio Basic Inc.) Each isolate was tested on a GC agar supplemented with 1 % of defined growth supplement containing 2-fold-increasing concentrations of antimicrobial agents. Plates were incubated in 5 % CO_2_ at 37 °C for 24 h followed by recording the antimicrobial susceptibility results. The production of β-lactamase enzyme was detected using nitrocefin discs.* N. gonorrhoeae *strain ATCC 49226 was used as a control. The criteria of reduced susceptible to cefixime and ceftriaxone is according to the WHO document (WHO, 2012[65]) where *N. gonorrhoeae* strains displaying reduced susceptibility to cefixime exhibit MICs of ≥ 0.25 μg/ml and *N. gonorrhoeae* strain displaying reduced susceptibility to ceftriaxone yield MICs of ≥ 0.125 μg/ ml.

### Whole cell protein extraction

The preparation of protein extracts was performed as previously described (Isarankura-Na-Ayudhya et al., 2010[24]; Nabu et al., 2014[40]). Briefly, the aforementioned eight-hour cultures of *N. gonorrhoeae* were chilled on ice and then harvested by centrifugation at 6,000 rpm for 10 min at 4 °C. Pellets were washed twice with cold PBS pH 7.2 and twice with cold Tris-sucrose pH 7.2 at 14,000 rpm for 10 min at 4 °C. Bacterial pellets were re-suspended in 400 ml of lysis buffer (7 M urea, 2 M thiourea, 4 % (w/v) CHAPS) containing 10 µl/mL protease inhibitors. Cells were disrupted using Branson sonifier (model 450) for 10 min on ice. The soluble fraction was collected by centrifugation at 14,000 rpm for 20 min at 4 °C. The total protein concentration was quantified by Bradford's method.

### Two-dimensional gel electrophoresis (2-DE)

Two-dimensional gel electrophoresis was performed using 2-D Electrophoresis System (GE Healthcare, USA) according to manufacturer's recommendations. Two hundred microgram of protein sample was adjusted to a final volume of 310 µL with rehydration buffer (8 M urea, 4 % (w/v) CHAPS, 0.002 % bromphenol blue) containing 2.8 mg/ml DTT, 2 % IPG buffer pH 3-10, and 12 μl/ml destreak. Proteins were separated in the first dimension using 13-cm pH 3-10 Immobilized pH gradient (IPG) strips (GE Healthcare). Isoelectric focusing (IEF) was carried out at 20 °C in Multiphor II Electrophoresis unit as follows: 50 V, 2 mA, 5 W, for 10 h; 300 V, 2 mA, 5 W, for 0.01 h; 3500 V, 2 mA, 5 W, for 1.30 h; 3500 V, 2 mA, 5 W, for 5 h; 500 V, 2 mA, 5 W, for 4 h. After IEF, strips were equilibrated twice for 15 min in equilibration solution (50 mM Tris-HCl, pH 8.8, 6 M urea, 30 % glycerol, 2 % SDS, bromphenol blue) containing 1 % DTT for the first step followed by 2.5 % iodoacetamide for the second step. The second dimension was carried out with 12 % SDS-polyacrylamide gels using the Hoefer SE600 system. Gels were run at 10 mA per strip for 30 min and then at 20 mA per strip until the dye front reached the bottom of the gel.

### Visualization of protein spots and differential analysis

Gels were stained with colloidal Coomassie brilliant blue G-250. Subsequently, gels were visualized and scanned by Image Scanner III (GE Healthcare). Differential and quantitative analysis were carried out using ImageMaster 2D Platinum version 7.0 (GE Healthcare) software tool. Differences in protein expression between control and treated sample were compared based on the ratio of percentage volume, where percentage volume is quantified by the sum of pixel intensities over the area of the individual spot divided by the total volume of all spots over the whole gel image multiplied by 100. Spot protein is considered to be differentially expressed, if it shows more than 1.2-fold increase/decrease compared to its appearance in control gel.

### MALDI-TOF Mass Spectrometry and protein identification

Mass spectrometry and peptide mass fingerprinting (PMF) analysis were carried out as previously described (Isarankura-Na-Ayudhya et al., 2010[24]). Protein spots of interest were manually excised from the gels. Spots were then soaked in 50 % methanol and 5 % acetic acid for overnight, subsequently digested with trypsin (Promega, USA). Protein extraction was carried on a Spot Handling Workstation (GE Healthcare) according to the manufacturer's protocols.

For protein identification, an aliquot of each mixture peptide was mixed with the solution of 10 mg/ml α-cyano-4-hydroxy-trans-cinnamic acid matrix solution in 66 % acetonitrile containing 0.1 % TFA (v/v) for MALDI-TOF analysis. Positive-ion MALDI mass spectra were recorded in the reflectron mode of a MALDI-TOF mass spectrometer. PMF search was performed using the BioTool 2.0 software (Bruker Daltonics) integrated with Mascot program (http:\\www.matrixscience.com) against NCBlnr database. The following parameters were considered for the searches: trypsin digestion with one missed cleavage permitted; fixed modification by carbamidomethylation of cysteines, and variable modifications by oxidation of methionine; and monoisotopic peptide masses. Search result scores that are greater than 80 are considered significant (*p*<0.05). The accuracy of the experimental to theoretical *p*I and molecular weight of proteins are also taken into consideration.

### Bioinformatics analysis

Subcellular localizations of identified proteins were predicted using PSORT-B, version 3.0 (http://www.psort.org). Biological functions of *N. gonorrhoeae* proteins were classified according to JCVI's CMR (https://www.ncbi.nlm.nih.gov/pubmed/11125067).

## Results

### Phenotypic characterization of N. gonorrhoeae

Phenotypes of *N. gonorrhoeae* were characterized based on their MICs to cefixime and ceftriaxone according to WHO document's criteria (WHO, 2012[65]). In the present study, two clinical isolates with reduced susceptibility to cefixime and/or ceftriaxone were selected. Namely, *N. gonorrhoeae* strain R1 exhibited reduced susceptibilities to cefixime (MIC 0.5 μg/ml), and ceftriaxone (MIC 0.125 μg/ml) while *N. gonorrhoeae* strain R2 displayed a reduced susceptibility only to cefixime (MIC 0.25 μg/ml). None of these produced the β-lactamase enzyme. Susceptibility patterns to other antibiotics are shown in Table 1(Tab. 1).

### Growth monitoring of N. gonorrhoeae upon ESC treatment

**Figure 1:**
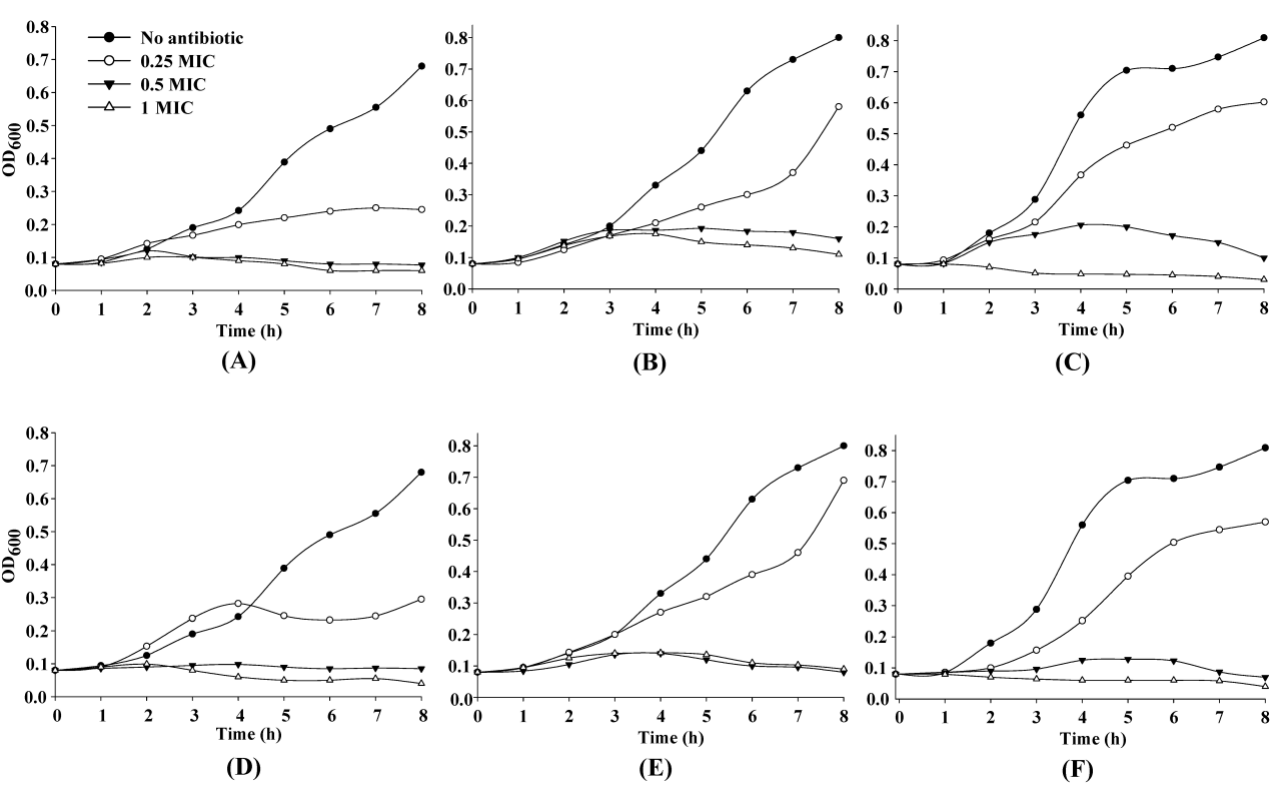
Figure 1: Growth characteristics of *N. gonorrhoeae* in the presence of various concentrations of extended-spectrum cephalosporins. *N. gonorrhoeae* ATCC 49226 (A and D), R1 (B and E), and R2 (C and F) were grown on GCB without (●), or with 0.25x (○), 0.5x (▼) or 1x (∆) MICs of ceftriaxone (A-C); and cefixime (D-F).

To investigate the effect of antibiotic stress on growth profiles of *N. gonorrhoeae*, bacterial cells were treated with 0.25x, 0.5x and 1x MICs of cefixime or ceftriaxone. Growth of ATCC 49226 was markedly reduced by approximately 50 % as compared to the control in the presence of 0.25x MIC of either cefixime or ceftriaxone starting from 5 h post-inoculation. Concentrations at 0.5x and 1x MICs of both antibiotics caused the growth arrest of ATCC 49226 over the 8 h of measurement time (Figure 1A(Fig. 1) and 1D(Fig. 1)). Growth kinetics of the R1 strain showed similar pattern in the presence of cefixime and ceftriaxone. Growth kinetics at 0.25x MIC of both antibiotics were shown to be markedly inhibited after exposure for 5 h and this is followed by gradual investigation of the increased turbidity at 8 h. Growth of this strain was found to be severely inhibited at 0.5x and 1x MICs (Figure 1B(Fig. 1) and 1E(Fig. 1)). For the R2 strain, 0.25x MIC of both antibiotics led to moderate growth reduction while 0.5x MIC caused severe growth inhibition. Addition of 1x MIC caused growth arrest and cell lysis at 4 h (Figure 1C(Fig. 1) and 1F(Fig. 1)).

### Identification of altered protein expression in susceptible strain upon exposure to sub-MIC doses of ESCs

**Figure 2:**
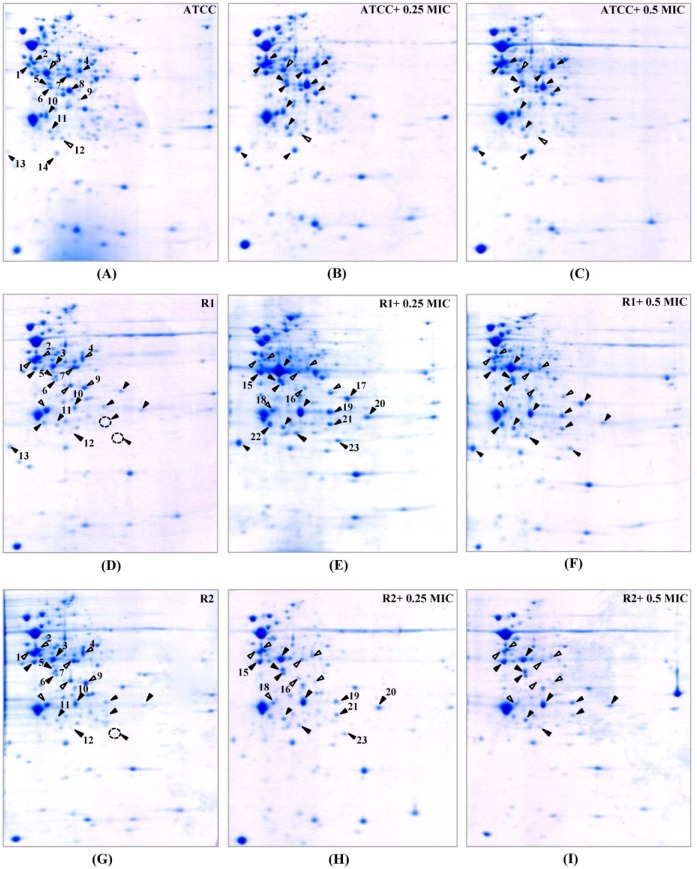
2-DE patterns of whole cell proteins of *N. gonorrhoeae *in response to ceftriaxone treatment. 2-DE of *N. gonorrhoeae *ATCC 49226 (A-C), R1 (D-F) and R2 (G-I) strains as cultured in GCB without and with 0.25x and 0.5x MICs of ceftriaxone. Spot numbers refer to proteins identified in Table 2. Black and white triangles represent up-regulation and down-regulation, respectively.

In order to study the cellular response of *N. gonorrhoeae* against ESC-induced stresses, ATCC 49226 were treated with 0.25x and 0.5x MICs of ceftriaxone and subsequently followed by evaluating relative differences of the protein expression via 2-DE gel image analysis. Our results revealed that 14 spot proteins (i.e. 12 proteins were up-regulated while 2 proteins were down-regulated) exhibited significantly different expressions upon ceftriaxone-treated condition (Figure 2A-C(Fig. 2)). These differentially expressed protein spots were then identified and classified based on their biological functions and subcellular localizations. Table 2(Tab. 2) shows that ceftriaxone-stress triggered proteins are present in various biological pathways and various cellular compartments. The majority of induced proteins (5 proteins) belonged to the class of energy metabolism. These included phosphopyruvate hydratase (i.e. an enolase), which was shown to be increasingly expressed in a dose-dependent manner (1.52 and 2.17-fold at 0.25x and 0.5x MICs, respectively); F0F1 ATP synthase subunit beta, acetate kinase and alcohol dehydrogenase, which showed increased expression at 0.25x higher than 0.5x MICs; and ubiquinol-cytochrome c reductase iron-sulfur subunit, which was increased by ~1.5-fold at both 0.25x and 0.5x MICs. Three proteins belonged to transport systems were up-regulated in response to ceftriaxone, which included two spot proteins of polyamine-binding periplasmic protein and one spot of amino acid ABC transporter periplasmic binding protein. Other up-regulated proteins consisted of glutamate dehydrogenase, fructose-1,6-bisphosphate aldolase, peptidyl-prolyl isomerase (Ng-MIP) and lipid modified azurin protein. Two proteins were down-regulated in the presence of ceftriaxone including elongation factor Tu (EF-Tu), which was found to be decreased by ~1.5-fold at both 0.25x and 0.5x MICs, and two-component system transcriptional response regulator OmpR was shown to be decreased in a dose-dependent manner.

**Figure 3:**
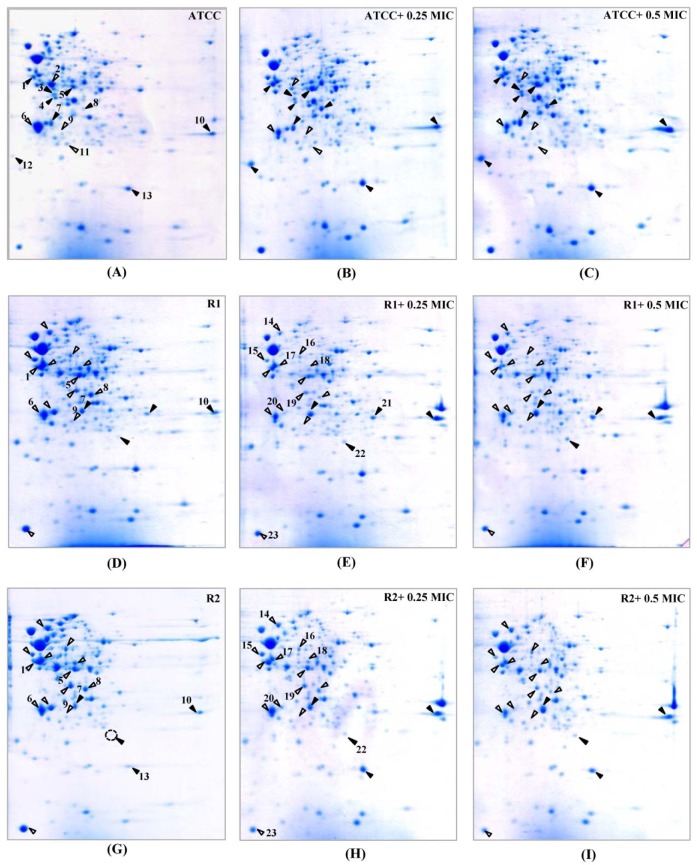
2-DE patterns of the whole cell proteins of *N. gonorrhoeae *in response to cefixime treatment. 2-DE of *N. gonorrhoeae *ATCC 49226 (A-C), R1 (D-F) and R2 (G-I) strains as cultured in GCB without and with 0.25x and 0.5x MICs of cefixime. Spot numbers refer to proteins identified in Table 3. Black and white triangles represent up-regulation and down-regulation, respectively.

Hence, we compared the cellular response of susceptible strain against ceftriaxone to those against cefixime, which is another type of ESC that is used for gonorrhoea treatment. As seen in Figure 3A-C(Fig. 3) and Table 3(Tab. 3), most induced proteins shared similar expression patterns to that of ceftriaxone-induced stress condition including enolase, acetate kinase, polyamine-binding periplasmic protein, fructose-1,6-bisphosphate aldolase, Ng-MIP, azurin, EF-Tu and OmpR. For proteins with different expression, RmpM was increased in a dose-dependent manner and 50S ribosomal protein L25 was increased by ~1.9-fold at both 0.25x and 0.5x MICs. Peroxiredoxin, a protein involved in detoxification, was decreased by ~1.5-fold at both 0.25x and 0.5x MICs and tetrahydrodipicolinate N-succinyltransferase (DapD), a protein involved in diaminopimelate biosynthesis, was decreased in a dose-dependent manner.

### Identification of altered protein expression in ESCs-reduced susceptible strains upon exposure to ESCs-induced stress conditions

Twenty-one proteins were differentially expressed upon exposure to sub-MICs of ceftriaxone as compared with the control cultures (shown in Table 2(Tab. 2) and Figure 2D-F(Fig. 2)). Five of these proteins showed increased expression resembled those found in the susceptible strain that comprises of the following: (i) proteins belonging to transport systems (i.e. two spot proteins of polyamine-binding periplasmic protein and one spot of amino acid ABC transporter periplasmic binding protein), (ii) proteins associated with virulence factors (i.e. Ng-MIP that was found to be increased by 4.12- and 2.94-fold at 0.25x and 0.5x MICs, respectively) and (iii) azurin (i.e. a protein involved in stress defenses where 2.12- and 2.43-fold increased in expression was found at 0.25x and 0.5x MICs, respectively). For proteins with different expression, all proteins belonged to the class of energy metabolism that were found afford decreased expression. Two proteins involving in the amino acid biosynthesis were found to be expressed in a different manner. Particularly glutamate dehydrogenase showed a decreased expression in a dose-dependent manner while cysteine synthase displayed an increased expression at 0.25x that was found to be higher than 0.5x MICs. For proteins belonging to the class of protein synthesis, it was found that elongation factors (EF)-Tu was up-regulated while EF-Ts was down-regulated. Moreover, proteins belonging to various functional classes showed increased expression in response to ceftriaxone, which included cell division (i.e. FtsZ and MinD), type IV pilus (i.e. PilT and RegF), transport and binding proteins (e.g. EtfB and aa-SBP_2) and regulatory protein (e.g. OmpR).

Furthermore, cellular responses of reduced susceptible strain to cefixime were investigated and compared to those with ceftriaxone treatment (Figure 3D-F(Fig. 3) and Table 3(Tab. 3)). Seventeen spot proteins were differentially expressed upon exposure to sub-MIC of cefixime, as compared to control cultures. We found that approximately half of the differentially expressed proteins shared a similar expression pattern to that of ceftriaxone-stress response including enolase, acetate kinase, GAPDH, fructose-1,6-bisphosphate aldolase, F0F1 ATP synthase subunit β, glutamate dehydrogenase, Ng-MIP, EF-Ts, RegF and MinD. For proteins with different expression, peroxiredoxin, dihydrolipoamide dehydrogenase (DLDH) and trigger factor showed decreased expression at 0.25x that was found to be higher than that of 0.5x MICs. DapD and F0F1 ATP synthase subunit α and 50S ribosomal protein L7/L12 were decreased in a dose-dependent manner. Lastly, RmpM was increased in a dose-dependent manner.

**Figure 4:**
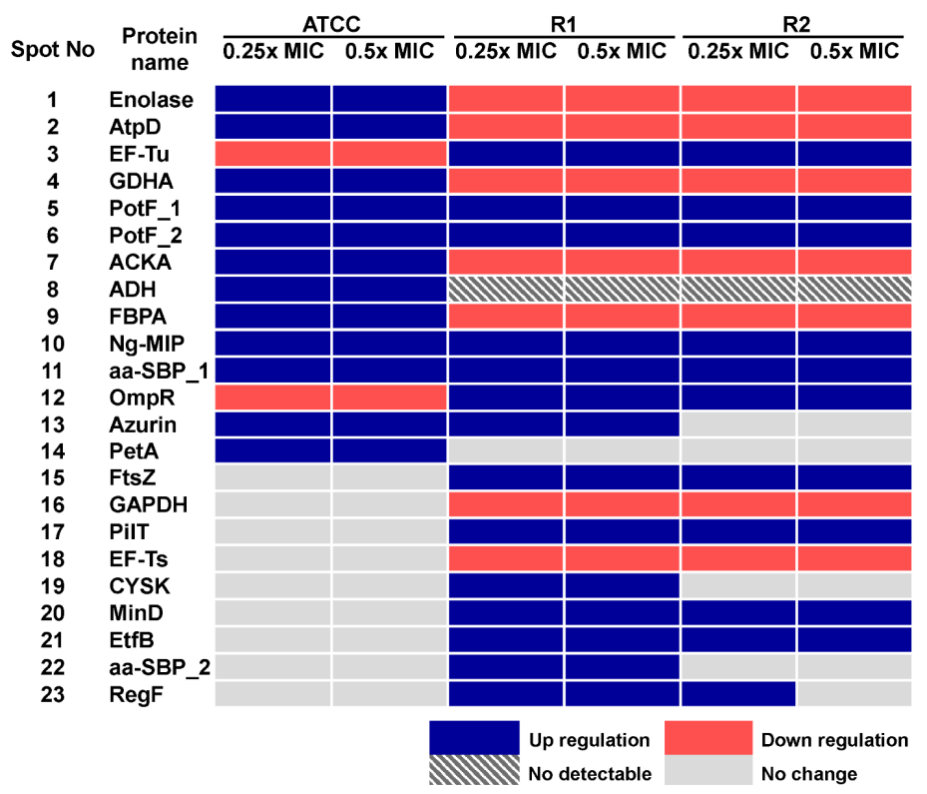
Figure 4: Summary of altered protein spots upon treatment with sub-MICs of ceftriaxone as assessed by 2-DE analysis

**Figure 5:**
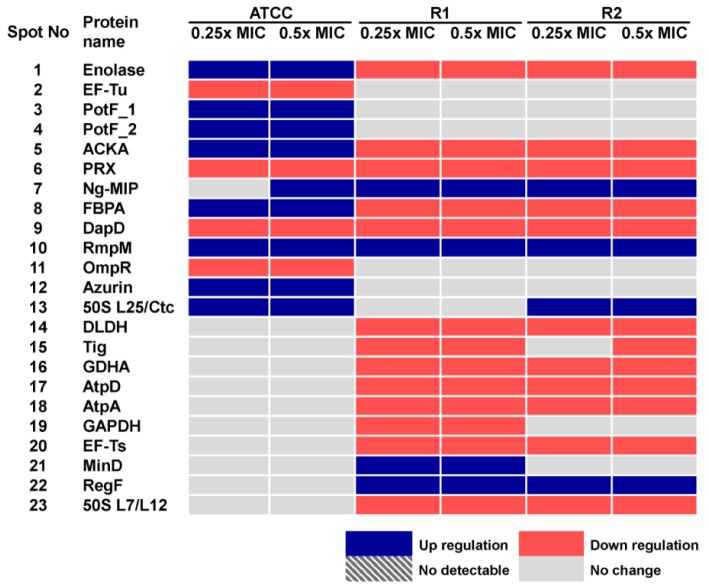
Figure 5: Summary of altered protein spots upon treatment with sub-MICs of cefixime as assessed by 2-DE analysis

Moreover, in order to investigate the consistency of protein expressions against ESC-induced stresses, proteomics profiling of R2 strain displaying reduced susceptibility to cefixime under ESC stresses was performed. Results showed that R2 strain showed 18 spot proteins with differential expressions in response to ceftriaxone treatment (Figure 2G-I(Fig. 2) and Table 2(Tab. 2)). Almost all differentially expressed proteins of R2 exhibited similar patterns as those of R1 strain with the exception of the RegF, which showed increased expression only at 0.25x MIC of ceftriaxone. Finally, proteomics profiling of the R2 strain under sub-MIC of cefixime was also investigated ( Figure 3G-I(Fig. 3) and Table 3(Tab. 3)). Again, we found that expressed proteins of the R2 strain were similar to the expression pattern of the R1 strain upon exposure to cefixime. Only one protein was differentially expressed, the 50S rRNA L25 exhibiting ~2-fold increase in expression upon exposure to cefixime. It should be noted that an increased expression of L25 under cefixime-stress in the R2 strain was also found in the susceptible strain. Figures 4(Fig. 4) and 5(Fig. 5) summarize the differential expression of proteins in exposure to sub-MICs of ceftriaxone and cefixime, respectively.

### Investigation of Ng-MIP expression upon exposure to other therapeutic antibiotics

**Figure 6:**
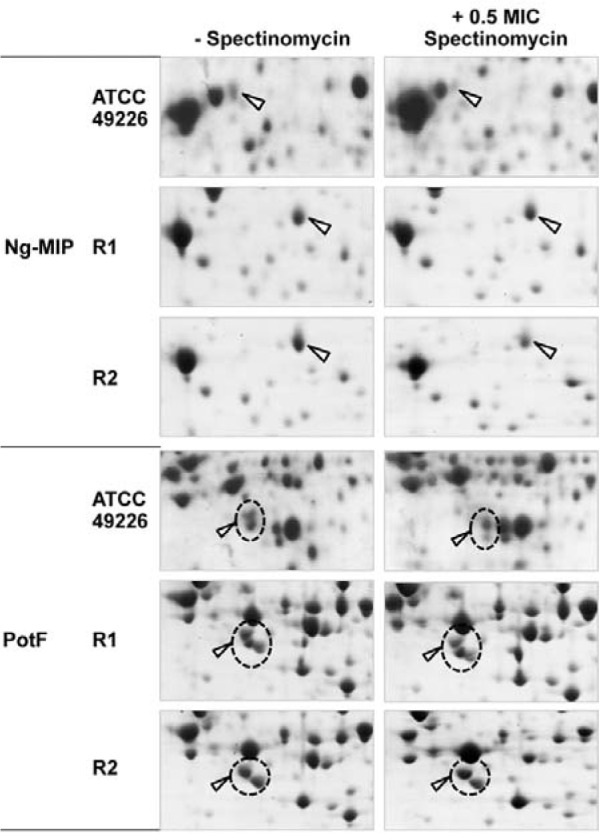
Enlarged partial 2-DE gels of selected proteins in response to treatment with 0.5x MIC or without spectinomycin in *N. gonorrhoeae* ATCC 49226, R1 and R2 strains as visualized by 2-DE

Since Ng-MIP was found to have an increased expression in both ESC-susceptible and ESC-reduced susceptible strains in ESC-induced stresses. Therefore, to address the question of whether the increased expression of Ng-MIP is a specific response of *N. gonorrhoeae* against ESC-induced stress, we investigated 2-DE gel of ATCC 49226, R1 and R2 strains upon exposure to antibiotics targeting the bacterial protein synthesis (e.g. spectinomycin and azithromycin). We found that there was no significant difference in the Ng-MIP expression in the presence or absence of antibiotics (Figure 6(Fig. 6)). Thus, our results suggested that the increased expression of Ng-MIP was a specific response to treatment of ESC or cell wall-targeting antibiotics.

Moreover, polyamine-binding periplasmic protein (PotF) that was found to yield an increased expression in both ESC-susceptible and ESC-reduced susceptible strains in ceftriaxone treatment was also analyzed. The PotF protein showed no significant alteration upon exposure to spectinomycin thereby implying that PotF directly responded to ceftriaxone.

## Discussion

Antimicrobial resistance in *N. gonorrhoeae* is a global public health problem and the greatest concern is the possibilities for the emergence of *N. gonorrhoeae *with decreased susceptibility and resistance to ESCs, which are the last remaining first-line treatment option for gonorrhea. This portends to an upcoming era of untreatable gonorrhoea. *N. gonorrhoeae* has a remarkable ability to develop resistance to previously and currently recommended antibiotics that are used for treatment. Therefore, it is essential to extensively study not only the primary resistance mechanism but also on the discovery of novel targets and novel antibiotics for effective treatment (Alirol et al., 2017[2]). Thus, proteomics profiling of soluble proteins of *N. gonorrhoeae *has been studied herein as to investigate secondary or non-target responses aside from those that are already known in regards to ESC resistance determinants. In the absence of antibiotics, susceptible strain (ATCC 49226) and two reduced susceptible strains (R1 and R2) exhibited a relatively similar growth pattern thereby suggesting that these reduced susceptible strains were not deficient in their biological fitness as caused by AMR mechanisms.

Moreover, protein profiles of susceptible and reduced susceptible strains were relatively similar in the absence of antibiotics; thus, cellular responses of these strains to any changes can be comparable without a bias from their own natural variation. Subsequently, cellular responses of susceptible and reduced susceptible strains to 0.25x and 0.5x MICs of ceftriaxone or cefixime were then analyzed by 2-DE. Although standard solubilizing agents as used herein for protein preparation led to lower achievement of membrane proteins, our 2-DE gel analysis could detect some proteins that are located in the membrane compartment, which were found to be significantly expressed under ESC stress. Therefore, further studies regarding the subcellular fractionation of samples prior to 2-DE is needed for investigating the effects of cell wall and membrane proteomes of gonococci in response to ESC treatment. We found that many differentially expressed proteins shared similar expression patterns between ceftriaxone and cefixime. Although some proteins showed specific response for each antibiotic, these results could be explained by the difference in their permeation properties. Cefixime has been proposed to diffuse into the periplasm by means of a different route from that of ceftriaxone; consequently, cefixime might trigger proteins or regulatory systems that are different from those of ceftriaxone (Unemo and Nicholas, 2012[60]). Furthermore, pertaining to the case of the ESC-reduced susceptible strain, we found that proteomics profiling of R1 and R2 strains were almost similar. This result indicated the consistency of our proteomics system, thus cellular responses of the R1 strain was used as a representative for discussion. Differentially expressed proteins found in both susceptible and reduced susceptible strains in response to ESC are proposed to be common adaptive mechanisms of gonococci in surviving under cell wall-active antibiotics. In addition, differentially expressed proteins uniquely found in reduced susceptible strains might be due to a secondary resistance mechanism of the reduced susceptible strain in overcoming the inhibition of cell wall synthesis by ESCs.

### Common adaptive responses between susceptible and reduced susceptible strains under ESC stresses

We firstly considered common adaptive mechanisms in response to ESC stress in both susceptible and reduced susceptible strains. Four proteins were increasingly expressed under ceftriaxone stress in both susceptible and reduced susceptible strains and this included polyamine-binding periplasmic protein (PotF), amino acid ABC transporter periplasmic binding protein (aa-SBP), macrophage infectivity potentiator (Ng-MIP) and azurin. Increased expression of these nutrient transport systems implies the adaptation of gonococci in surviving in nutrient-limited conditions, such as that in host cells or in adverse conditions of antibiotic treatment. The increased expression of the amino acid transport system has been documented in *Staphylococcus aureus* in response to sub-MIC of thioridazine, a compound that has been reported to disturb the peptidoglycan synthesis (Thorsing et al., 2013[52]). *N. gonorrhoeae *displays a single polyamine transporter system (PotFGHI) with three putative periplasmic polyamine binding proteins that are encoded by *potF1 *(NGO0206), *potF2* (NGO1253), and *potF3 *(NGO1494). Two spot proteins of PotF as visualized by 2-DE had peptide mass fingerprinting that matched the profile of *potF3*. PotF was found to have increased expression only under ESC-induced stress. Polyamines, such as putrescine, spermidine and spermine, are polycationic molecules that are necessary for cell growth, DNA and RNA binding and resistance to oxidative stress (Tabor and Tabor, 1985[49]). Polyamines have been reported to be antioxidant molecules that defend against oxidative stresses induced by hydrogen peroxide, superoxide radicals and antibiotics (Tkachenko et al., 2001[53], 2012[54]). Additionally, polyamines have been suggested to increase the antibiotic resistance in *E. coli.* Particularly, polyamines can reduce the flux of cephaloridine via OmpF of *E. coli* by binding inside the opened channel thereby resulting in closed channels (Dela Vega and Delcour, 1996[14]; Iyer and Delcour, 1997[26]). In contrast, these circumstances are quite different from that found in *N. gonorrhoeae* in which polyamines were found to significantly increase gonococcal resistance to polymyxin B and host-derived antimicrobials cathelicidin LL-37 and complement-mediated killing by normal human serum but did not alter the susceptibility of β-lactams (Goytia and Shafer, 2010[19]). Therefore, increased expression of PotF under ESC treatment might aid gonococci to evade the innate host defense in human and might be associated with the adaptation of gonococci to survive under oxidative stress as induced by antibiotics. The latter suggestion is in agreement with an increased expression of azurin, which is a protein involved in the defense against oxidative stress.

Furthermore, our results significantly emphasized that the sub-inhibitory concentration of antibiotics can enhance the expression of bacterial virulence factor as indicated by the increased expression of azurin and Ng-MIP. In particular, azurin has been reported to be involved in gonococcal virulence factor by facilitating the microorganism to survive within cervical epithelial cells (Wu et al., 2005[68]). In the meanwhile, Ng-MIP is a lipoprotein that is anchored to the outer membrane and is required for persistence of *N. gonorrhoeae *in the macrophage and for protection against the macrophage-mediated killing while exerting no influence to invasion and survival in other cells (Leuzzi et al., 2005[32]). Ng-MIP has been reported to exhibit a peptidyl prolyl *cis-trans* isomerase (PPIase) activity, which functions to catalyze the *cis/trans* isomerization of prolyl peptide bonds in the proline-containing polypeptide. PPIase plays an important role in protein folding, reactivation of denatured proteins and protein synthesis (Kromina et al., 2008[29]). Gonococci contains five PPIase comprising of PpiA and PpiB of the cyclophilin family, Mip and SlyD of the FKBP family and PpiD of the parvulin family (Du and Arvidson, 2006[15]). In this study, it was found that Ng-MIP was significantly upregulated, particularly in ESC-reduced susceptible strains and under ESC treatment. It was also found that Ng-MIP was shifted to a more basidic *p*I in both ESC-reduced susceptible strains. Furthermore, the protein expression pattern of Ng-MIP for 4 ESC-reduced susceptible strains and 4 ESC-susceptible strains without antibiotics was compared by 2-DE gels. However, we could not find any correlation between the *p*I shift of Ng-MIP and the antimicrobial resistance phenotype, which may be due to the limited number of the reduced susceptible strain (data not shown). Previous study found that amino acid substitutions in Ng-MIP (i.e. Glu-160-Lys and/or Ser-49-Gly) could be detected in clinical isolates (Starnino et al., 2010[46]). These mutations might result in *p*I shift of Ng-MIP that was also detected in our 2-DE experiment. Even though these amino acid substitutions did not affect its enzymatic activity, Ng-MIP mutation regarding ESC-reduced susceptibility should be further investigated in a larger number of *N. gonorrhoeae* isolates. Moreover, the expression of this protein is found to be a unique response against ESC treatment (i.e. both ceftriaxone and cefixime) without eliciting any significant change under other antibiotic classes (e.g. spectinomycin and azithromycin) thereby suggesting that this protein is highly sensitive to antibiotics inhibiting cell wall synthesis. Although the association of MIP or FKBP family with antibiotics response has never been documented, we proposed that upregulation of Ng-MIP might be an adaptation or a tolerance mechanism of gonococci in response to ESC stress, perhaps similar to the intracellular survival in macrophage. It should be noted that PrsA, a PPIase enzyme in the parvulin family, in *Bacillus subtilis* is required for PBP folding and cell wall biosynthesis (Hyyryläinen et al., 2010[23]) while PrsA from *Staphylococcus aureus* was found to be upregulated under treatment with cell wall-active antibiotics (Kuroda et al., 2003[30]; Utaida et al., 2003[61]). Moreover, PPIase proteins from *Acinetobacter baumannii* was found to have significantly increased expression in β-lactam resistant strain (Vashist et al., 2010[62]). Thus, the association of Ng-MIP with β-lactam response or cell wall biosynthesis should be further investigated in more details. It should be emphasized that MIP has been identified as an important virulence factor in various pathogens such as in *Legionella pneumophila, Trypanosoma cruzi, Escherichia coli *and *Salmonella enterica *(Cianciotto et al., 1990[11]; Horne et al., 1997[22]; Justice et al., 2005[27]; Moro et al., 1995[39]). Moreover, this protein has been suggested as a potential target for anti-bacterial and anti-parasite infections (Bharatham et al., 2011[5]; Unal and Steinert, 2015[55]). Similarly, Ng-MIP is an important virulence factor for pathogenesis expressed in all gonococcal isolates. Taken together with our results this suggests a significant association with the antibiotic stress response. Thus, Ng-MIP is proposed to be a potential target for antibiotic therapy for use as a dual antimicrobial treatment regimen (Reimer et al., 2016[43]).

Additionally, Rmp was found to have increased expression particularly for cefixime treatment in both susceptible and reduced susceptible strains. This protein plays roles in the formation, stabilization and/or operation of PorB porin and in cross-linking the outer-membrane proteins to the peptidoglycan. Thus, the upregulation of this protein particularly in cefixime treatment might support the previous suggestion that cefixime is fluxed in/out of gonococcal cells in a different manner than that of ceftriaxone (Unemo and Nicholas, 2012[60]; Zhao et al., 2009[71]). Tetrahydrodipicolinate N-succinyltransferase (DapD) showed decreased expression in response to cefixime stress. DapD is an enzyme involved in the lysine/diaminopimelic acid biosynthetic pathway. The meso-diaminopimelic acid (meso-DAP) is a pentapeptide that is essential for cross-linking peptidoglycan chains. The downregulation of this enzyme might indicate the influence of ESCs on bacterial cell wall synthesis. In support of this idea, a recent study has shown that β-lactams not only inhibit the PBP activity but also deplete the biosynthesis of the peptidoglycan precursor, thus resulting in enhanced bactericidal activity (Cho et al., 2014[10]).

### Differential expression profiles between susceptible and reduced susceptible strains in response to ESC stresses

Proteins involved in energy metabolism and cell division process appear to be differentially expressed in responses to ESC stress between susceptible and reduced susceptible strains. Alteration of physical catabolism as influenced by antibiotics is a common mechanism found in several bacteria (Lima et al., 2013[34]). Susceptible strain showed an increased expression of enolase (e.g. ACKA and AdhA) thereby suggesting that the ESC-susceptible strain utilized the metabolic fermentation under ESC-stress. It should be noted that AdhA is activated by PerR, a H_2_O_2_-responsive regulator (Wu et al., 2006[69]). Although the role of gonococcal AdhA in oxidative stress is unknown, this protein was reported to be involved in resistance to oxidative stress in other organisms (Echave et al., 2003[16]). A plausible explanation is that AdhA maintains NADPH/NADP generation, which is required for the maintenance of intracellular redox balance. Consequently, an increased expression of proteins in the electron transport chain (ETC) (i.e. F0F1 ATP synthase subunit beta (AtpD) and ubiquinol-cytochrome C reductase iron-sulfur subunit (PetA)) could result from an increased level of NADPH/NADP.

**Figure 7:**
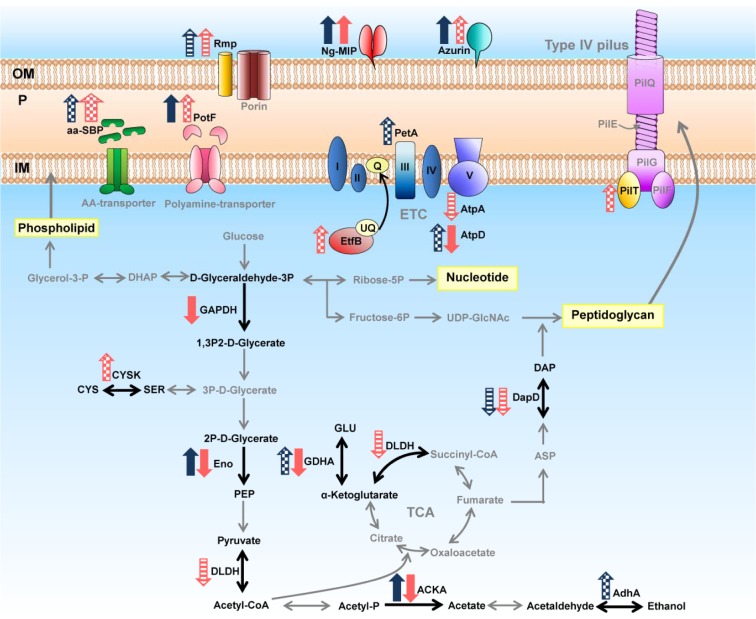
Altered expression of proteins involved in energy metabolism and found in cell envelope in response to treatment with sub-MIC of extended-spectrum cephalosporins. Dark blue arrows and light red arrows represent expressed proteins of the susceptible strain and of the reduced susceptible strain R1 in response to both cefixime and ceftriaxone, respectively. Crosshatched arrows represent expressed proteins in response to ceftriaxone treatment. Striped arrows represent expressed proteins in response to cefixime treatment.

On the other hand, ESC-reduced susceptible strain showed a decreased expression of proteins involved in metabolism (i.e. enolase, acetate kinase, glyceraldehyde 3-phosphate dehydrogenase, F0F1 ATP synthase subunit beta and glutamate dehydrogenase). These results implied that when reduced susceptible strain is under adverse condition, it reduces the general metabolism that is unnecessary for strict survival. The concise model for energy metabolism and cell envelope of *N. gonorrhoeae* in response to ESCs is illustrated in Figure 7(Fig. 7). Interestingly, we found that FtsZ and MinD was upregulated in ESC-reduced susceptible strains under ESC stress. These proteins play an essential role in cell division and cell shape. Filamenting temperature-sensitive mutant Z (FtsZ) is an initial protein that localizes to the incipient division site to form a ring-like structure (the Z-ring). Subsequently, cell division process carries out by recruiting the other cell-division proteins to form the divisome (i.e. constriction of the cell membrane) and splitting of the daughter cells. The precise placement of the Z-ring formation is accomplished by the MinCDE system as to ensure that division occurs at the midcell (Lutkenhaus, 2007[37]). Our results indicated that the increased expression of FtsZ and MinD might be an additional resistance mechanism to overcome the inhibition of cell wall synthesis by cephalosporins. Thus, cell division can occur without additional peptidoglycan synthesis thereby resulting in a reduction of the effectiveness of antibiotics. Our finding is in good agreement with previous report on *E. coli* resistance to amdinocillin (Vinella et al., 1993[63]). Several recent studies have demonstrated that bacterial cell division process, in particular the FtsZ protein, is a promising target for novel antibacterial development (Awasthi et al., 2011[4]; Schaffner-Barbero et al., 2012[44]). Moreover, results from whole-genome sequencing revealed that an additional R251H substitution in the ftsX gene is implicated in cephalosporin resistance (de Curraize et al., 2016[13]). Thus, our findings together with previous studies speculate that FtsZ is a potential target for drug development particularly for antimicrobial-resistant bacteria (Haydon et al., 2008[21]; Stokes et al., 2013[47]; Sun et al., 2014[48]).

Furthermore, we found that ESC-reduced susceptible strain displayed increased expression of PilT, which is a protein implicated in the pilus retraction. PilT plays many roles in gonococcal pathogenesis such as DNA transformation, twitching motility and host cell interaction (Merz and So, 2000[38]; Wolfgang et al., 1998[67]). Again, as mentioned above, our findings revealed that the sub-inhibitory concentration of antibiotics triggered the expression of gonococcal virulence factor that is important for the pathogenesis of gonorrhoea such as azurin, Ng-MIP and also PilT. Such finding is supported by several studies showing that sub-lethal dose of antibiotic acts as a signal to activate bacterial adaptation, communication (such as quorum sensing) and virulence factor (Linares et al., 2006[35]; Raivio, 2005[42]; Shen et al., 2008[45]). This circumstance cautions against the usage of antibiotics for treatment in which the sub-inhibitory concentration of antibiotics, which can appear in the host after treatment or even in case of misuse of the antibiotic (i.e. such as improper dosage), not only gives a selective pressure for bacteria to develop antibiotic resistance and treatment failure but also increases the pathogenesis of disease.

## Conclusion

The present study explores the protein expressions of a wide range of biological processes of *N. gonorrhoeae* in response to the treatment of extended-spectrum cephalosporin antibiotics. Increased expressions of nutrient transport systems were identified in both ESC-susceptible and ESC-reduced susceptible strains thereby indicating the adaptation of gonococci to survive under adverse conditions. Moreover, ESCs-reduced susceptible strains uniquely increased the expression of proteins that are involved in cell division (e.g. FtsZ and MinD) under ESC stress. The increase of cell division process is supposed to be an additional resistance mechanism of the reduced susceptible strain to counterbalance the cell wall synthesis inhibition by ESCs. Significantly, we revealed that sub-inhibitory concentration of antibiotics could trigger many gonococcal virulence factors such as azurin, Ng-MIP and PilT. Our findings also emphasize the importance of antibiotic usage (i.e. at sub-inhibitory concentrations) not only to provide a selective pressure for bacteria to develop antibiotic resistance but also to increase the pathogenesis of the disease. Overall, this broad view study on the global cellular response of gonococci to antibiotics is expected to provide a comprehensive understanding of physiological changes in gonococci in response to antibiotics and also to explore potential targets for further drug development.

## Conflict of interests

The authors have declared that no competing interests exist.

## Acknowledgements

S. N. is a Ph.D. student financially supported by the Thailand Research Fund through the Royal Golden Jubilee PhD Program (grant no. PHD/0195/2550) under the supervision of V. P. Partial support is also acknowledged from the Office of the Higher Education Commission and Mahidol University under the National Research Universities Initiative and annual research budget of Mahidol University (B.E.2557-2559).

## Figures and Tables

**Table 1:**
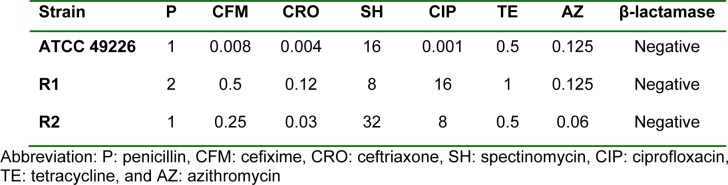
Minimal inhibitory concentrations (*μ*g/ml) of *N. gonorrhoeae* isolates

**Table 2:**
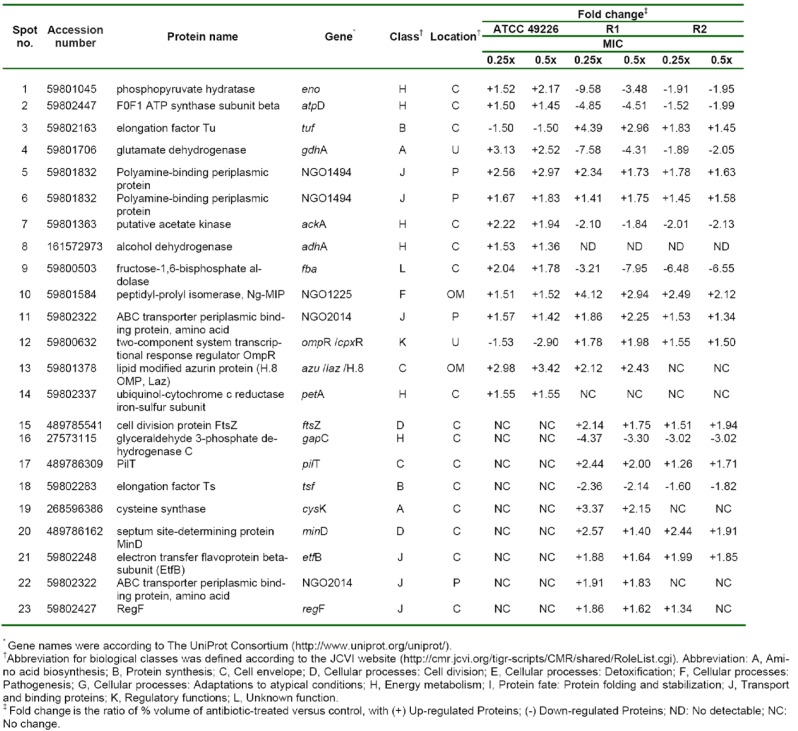
Proteins differentially expressed of *N. gonorrhoeae* in response to ceftriaxone treatment

**Table 3:**
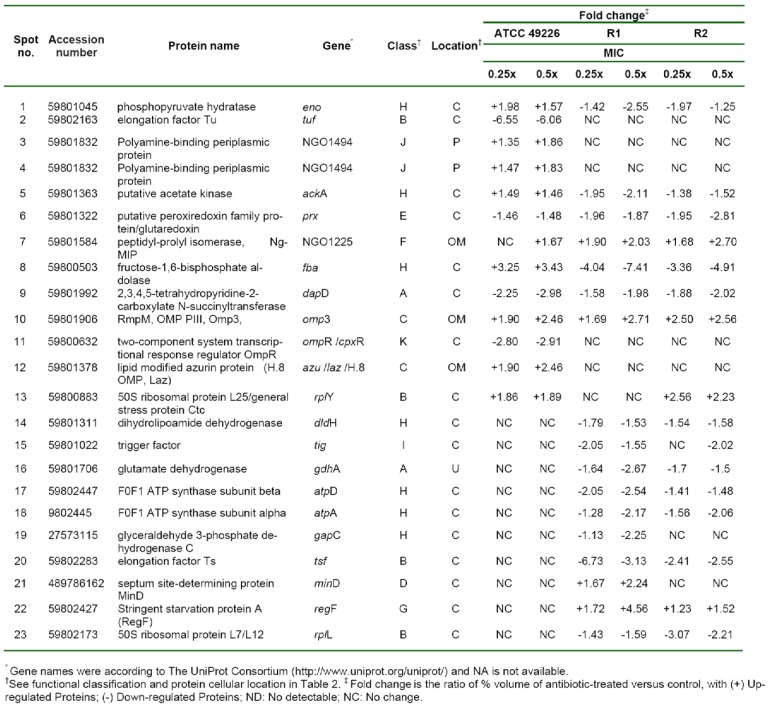
Proteins differentially expressed of *N. gonorrhoeae* in response to cefixime treatment
